# The role of trust in global health research collaborations

**DOI:** 10.1111/bioe.12536

**Published:** 2018-11-27

**Authors:** Angeliki Kerasidou

**Affiliations:** ^1^ The Ethox Centre University of Oxford Oxford United Kingdom of Great Britain and Northern Ireland

**Keywords:** collaboration, global health research, professional ethics, reliance, trust

## Abstract

Collaborations in global health research are on the rise because they enhance productivity, facilitate capacity building, accelerate output and make tackling big, multifactorial research questions possible. In this paper, I examine the concepts of trust and reliance in scientific collaborations in general, but also in the particular context of collaborations in global health research between high‐income countries and low‐and‐middle income countries (LMIC). I propose and defend the argument that given the particular characteristics of collaborations and demands of trust relationships, reliance is a better relational mode for successful collaborations. Although reliance can be difficult to establish in situations where asymmetry of power exists, trust should not be the only relational mode available to LMIC researchers because of the type of vulnerability it introduces to the relationship. I conclude that the promotion of good collaborations requires addressing the power imbalances between partners, and establishing an even playing field in global health research.

## 1 INTRODUCTION

In this paper I examine the role of trust in forming and maintaining scientific collaborations in global health research. Global health research provides an especially interesting context in which the role of trust in collaboration can be examined due to the particular dynamics between the collaborating partners.

Global health research is an emerging field of scientific enquiry that often relies on collaborations between researchers from high‐income countries (HIC) and low‐and‐middle income countries (LMIC).^1^ LMIC still carry the highest burden of disease globally.^2^ Since the 1990s, when the Global Forum for Health Research first adopted the term ‘90/10 gap’ to highlight the inequality in the distribution of health research funds (because less than 10% of worldwide resources are devoted to health research in developing countries where 90% of preventable deaths worldwide occur), much attention has been given to this issue. National and international funders, such as The Bill and Melinda Gates Foundation,^3^ the National Institutes of Health (NIH),^4^ the Research Council in the U.K.^5^ and the Wellcome Trust,^6^ have dedicated funds to global health research. These funds have resulted in a significant increase in interest from HIC researchers to tackle diseases and conditions such as malaria, tuberculosis, HIV‐AIDS and malnutrition,^7^ and a desire among HIC researchers to work with colleagues from LMIC. The aim of these initiatives is twofold: first, to promote and accelerate the discovery of pharmaceuticals and therapeutics; second, to help build research capacity in LMIC, so local researchers will be able in the future to set their own agendas to pursue innovative and locally relevant health‐related research without the need of an HIC partner.

HIC and LMIC collaborations do not always work as intended. It has been observed that these types of collaborations are often structured in ways that favour HIC researchers more than their counterparts in LMIC.^8^ For example, research partners from LMIC often find themselves in the role of ‘glorified field workers’, providing the samples and data, but less involved – if at all – in designing the research and setting research agendas.^9^ This relationship curtails LMIC researchers’ opportunities to establish themselves in their field and to pursue their own research interests, and results in the perpetuation of the situation it is seeking to address: a situation where LMIC researchers remain dependent on their HIC counterparts for funding and research opportunities.

In 2013, the Council on Health Research for Development (COHRED) issued a report entitled *Where there is no lawyer: Guidance for fairer contract negotiation in collaborative research partnerships*.^10^ The aim of this document is to assist researchers from LMIC to achieve better and fairer collaborations with their HIC partners. COHRED acknowledges that ‘sporadic attempts’ to level the scientific playing field between HIC and LMIC, as well as calls for HIC researchers to abide by the principles of fairness when entering into partnerships with LMIC researchers have had limited success. By issuing this guidance, COHRED seeks to ‘shift the locus of control of research benefits to the LMIC partner’,^11^ and ensure that LMIC researchers do not have to trust to their richer colleagues’ ‘good will’. Trust is important, says COHRED, but more is needed for good and fair collaborations. So, if LMIC researchers cannot rely on trust for fair collaborations, where should they turn?

In this paper, I examine the concepts of trust and reliance in scientific collaborations in general, but also in the particular context of collaboration in global health research between HIC and LMIC. I propose and defend the argument that, given the particular characteristics of collaboration and demands of trust relationships, reliance is a better relational mode for successful collaborations. Although reliance can be difficult to establish in situations where asymmetry of power exists, trust should not be the only relational mode available to LMIC researchers because of the type of vulnerability it introduces to the relationship. I conclude that the promotion of good collaborations requires addressing the power imbalances between partners, and establishing an even playing field in global health research.

The goal of this paper is not to offer a new account of trust or reliance but rather to extend discussion already in the literature to bear upon the context of collaborations in global health research.

## 2 WHAT IS A COLLABORATION?

The existing consensus is that collaborations are good and ought to be promoted. It is argued that they are an efficient and effective way of answering scientific questions, and solving problems. Collaborations enhance research productivity, facilitate knowledge generation and knowledge transfer, and make tackling big, multifactorial research questions possible (e.g., in genomic epidemiology research). Particularly in global health research, collaborations are seen as an effective way of addressing global health disparities and building research capacity in LMIC.^12^ For these reasons, numerous initiatives have been set up to encourage collaborative research, not only amongst individual researchers but also institutions in global health research. Funders have created specific funding streams for collaborative projects,^13^ and governments have launched schemes to encourage collaboration between HIC and LMIC.^14^

Although there is little argument about the need for and the value of collaborations, defining what counts as one has proven more difficult. In everyday language we often use ‘collaboration’ to describe any type of partnership where two or more actors (e.g., individuals, groups or institutions) come together to share information and tools, communicate and interact with each other. But different partnerships require different levels of sharing, communicating or interacting. Consider, for example, two researchers exchanging genomic data to pursue their different scientific projects, and contrast this with another case where two researchers come together to investigate the genetic underpinnings of severe malaria. Although both cases require some level of sharing, interaction and communication between the actors involved, the extent and the degree required differ.

A number of scholars have tried to identify the specific characteristics of collaborations.^15^ According to Hord, collaboration is a relationship that involves shared authority and responsibility for the planning, implementation and evaluation of a joint effort.^16^ Similarly, Mattessich, Murray‐Close and Monsey define a collaboration as the coming together of two or more autonomous actors in order to fulfil a common mission that requires comprehensive planning, and communication.^17^ What distinguishes collaborations from other types of partnerships is the presence of a common goal, and convergence regarding plans of action and methods used.^18^ Collaborations are non‐hierarchical structures where the division of labour is based on capacity and expertise rather than on functions or titles.^19^ Sharing of decision making and of responsibility is fundamental for this type of partnership. Participation in the decision‐making process ensures a higher degree of investment from all members, and also greater commitment to the successful completion of the project. The non‐hierarchical structure of collaboration also means that establishing rules of engagement and of problem solving, as well as delegating responsibilities, are tasks that are approached in a collective and democratic manner. Input and approval are sought by all collaborating partners.

The specific characteristics of collaborations necessitate the exercise of certain traits and attitudes. Transparency, honesty in communication, appreciation of each other's positions and synergy are all listed as important characteristics in non‐hierarchical structures.^20^ Transparency facilitates a collective awareness of the project, its structure, strengths and weaknesses, and promotes collective ownership. Honesty in communication allows for the free flow of information and exchange of ideas, but also for the expression of concerns and worries. Understanding each other's positions and particular circumstances is crucial, as it helps with setting expectations at the right level, anticipating problems and identifying areas where conflict may arise. Also, the drive and desire to achieve the common goal, and recognition of the partners’ interdependence in fulfilling this aim means that synergy – the attitude that ‘something greater can always emerge out of a process or interaction’^21^ – rather than reciprocity^22^ is what drives such partnerships.^23^

Many scholars have argued that what underpins successful collaboration is a relationship of trust between the partners.^24^ D'Amour notes that ‘the term collaboration conveys the idea of sharing … in a spirit of harmony and *trust’*.^25^ I would like to turn now to this notion, and ask whether trust is important and necessary for collaborations.

## 3 TRUST

There are three main characteristics that describe trust relationships: (a) trust can only be conferred by the trustor, and it cannot be demanded by the trustee; (b) the trustor believes that the trustee has good will towards him or her; and (c) the trustor assumes a participant's stance, namely they make themselves vulnerable to the trustee as they acknowledge and accept that the trustee can decisively affect the outcome of the entrusted action.^26^ These characteristics explain and validate feelings of gratitude, when trust is confirmed, and of betrayal, when trust is broken. It is important to underline that vulnerability is not a necessary characteristic of the person who is trusting, but a relational property that emerges from the act of trusting. When I confide a secret to my friend and I ask him not to reveal it, I acknowledge that he has the power to either confirm my trust – which will make me feel grateful – or to ignore my request – making me feel betrayed. My vulnerability arises because I have no assurances, other than those bestowed by the trusting relationship, to protect me from his decision. His having good will towards me means being ‘directly and favourably’ moved by the thought that I am counting on him.^27^ It is this assumption or belief that a person has ‘good will’ towards me that justifies entering a position of vulnerability.^28^ It follows that without this belief or assumption my decision to trust him would be unreasonable.

It often takes time to build trust, and when broken, it is difficult to restore. As Baier notes:

‘Trust me!’ is for most of us an invitation which we cannot accept at will – either we do already trust the one who says it, in which case it serves at best as reassurance, or it is properly responded to with, ‘Why should and how can I, until I have cause to?’^29^

Being trustworthy means giving others reasons to believe that one can be counted on. Yet demonstrating trustworthiness moves beyond the mere observation of rules and regulations as a tactic to avoid punishment or penalties.^30^ Being trustworthy means having an attitude of good will towards the trustor by being responsive to the trustor's dependency upon the trustee.^31^

It is not immediately obvious why trust is necessary in collaborations. Ideally, people would not want to enter into a relationship which renders them vulnerable and entirely dependent on their collaborator's good will to counterbalance their vulnerability. Trust can be justified if the trustor has sufficient evidence of the trustee's good will – but it takes time to amass this evidence. One might trust a collaborator if one has known them for a long time and has come to believe that they are trustworthy, i.e., they are not only conscientious but also have good will towards one. In such a situation, trust can be a sufficient reason to enter into a collaborative partnership. However, not all collaborations are established between longstanding partners. When considering a collaboration with a new partner, the partner's reputation might be an indication of their trustworthiness; but again, it is not obvious why anyone would choose to become vulnerable to another person, on the basis of what a third party says about them. So, although in certain circumstances trust can be a sufficient reason for collaboration, it is not a necessary one.^32^

Researchers form collaborations to achieve goals that would be impossible to reach by working alone. They seek collaborations with colleagues who are interested in answering the same questions, and who also have the knowledge and skill to achieve it. A relational mode that seeks partners based on these qualities, rather than on the presence of good will, might be more appropriate for research collaborations. Could reliance provide a better relational mode for collaborations?

## 4 RELIANCE – A NECESSARY COMPONENT OF COLLABORATIONS

The terms ‘reliance’ and ‘trust’ are often used interchangeably but a closer inspection reveals that there are significant differences between them. Reliance describes a relationship where the involved parties come together through a process of rational exercise that aims at minimizing the risks and maximizing the benefits of the relationship, and one which distributes legitimate rights to the partners involved. Rules of engagement are clearly defined, and partners are open regarding their goals and aims. A clear system of accountability is put in place^33^ and paths of communication are established. Everybody knows what is expected of them, and what their role in the relationship is. Partners are required to follow the rules agreed upon, and their adherence to the rules is regulated by policies and penalties. As a relationship mode, reliance is one based on ‘reasonable expectations’ and ‘proven capability’.^34^

The crucial difference between a relationship of reliance and one of trust is that reliance does not require the adoption of a participant stance towards the other person. In other words, one does not need to take the risk of having one's trust betrayed or confirmed by acting on the belief that the other person has good will towards one, and that this good will forms a reason for fulfilling the trusted action.^35^ One only needs to be convinced that the other person has enough self‐interested reasons to reliably fulfil the action. These reasons can take different forms. It can be, for example, because the penalty for failing to keep his or her end of the deal would be too high, or because he or she wants to initiate a reciprocal relationship (prisoner's dilemma). The idea that researchers are motivated by self‐interested reasons to behave collaboratively, and that self‐interest plays a moderating role in reducing risk in such relationships is not new, and has been defended by a number of scholars.^36^

Presence of self‐interested reasons does not eliminate all risk in collaborations. Partners can still expose themselves to harm or wrong, but the harm or wrong that results from a reliance relationship would not make the harmed party feel betrayed. I rely on my co‐author to contribute to the writing of a manuscript. If she fails to fulfil her duties as a co‐author she will incur costs. I will remove her from the author list and might decide not to collaborate with her again. I might even share my experience with other colleagues, which will lead to reputational damage for her. When my collaborator reneges on her duty to keep her part of the deal, I am harmed: the manuscript will take longer to complete, or in the worst case I might even have to abandon this project. I may feel upset and annoyed by her behaviour; I do not, however, feel betrayed – as I would feel if a friend had let me down.^37^ This is because I would not expect my collaborator to (necessarily) reflect on my dependence on her, and actively and positively engage with this fact. As Jones explains, reliance and reliability is 'depend[ing] on each other in the sense that the success of our action is vulnerable to the other's choice of action, and often enough we recognize this, but we do not depend on the other responding to that dependency'.^38^

There is an affective element in trust, which is not present in relationships of reliance, and I maintain that it is this lack of emotional involvement that makes reliance more suited to scientific collaborations. A relationship of reliance is based on proven capacity and reasonable expectations. Scientists seek out collaborations in order to create new knowledge and to explore innovative ideas. This joint creation and exploration involves bringing diverse scientific fields together, sharing tools and resources, increasing the visibility of their work, and learning from each other.^39^ Proven capacity, along with reasonable expectations of deliverables within the parameters of expertise and stated research goals, seem to be the basic criteria on which collaborators are chosen.^40^ Mouzas et al., discussing the distinction between trust and reliance in the business context, observe that ‘whilst trust is associated with the acceptance of dependency and risk, reliance introduces an institutionalized standard to reduce risks’.^41^ Again, this distinction reflects the requirement within a collaboration to set clear rules of engagement in a democratic manner, in order to ensure acceptance from all members. When a group of researchers decides to collaborate by pooling resources and expertise to achieve a common goal (e.g., the discovery of a new vaccine), it would be fundamental to set the parameters of the collaboration in such a way as to minimize risks for all, maximize benefits and increase the possibility of successful completion of the project. In recent years, collaboration prenuptials, legal documents where agreements regarding data ownership, authorship and deliverables are spelled out, have been suggested as an efficient way to ensure and promote successful collaborations in research.^42^

So far, through an examination of collaborations, trust and reliance, I have argued that whereas trust can be a sufficient reason for collaboration, reliance presents as a more appropriate relational mode to guide such partnerships. Reliance is premised on self‐interest rather than good will, requires less time to be established between partners, and can be achieved by setting clear rules of engagement and systems of accountability. In research collaborations there is always the possibility that things might go wrong and that people might renege on their responsibilities. Therefore, collaborations based on proven capacity and reasonable expectations, rather than on beliefs of good will seem both reasonable and appropriate. What remains to be examined is whether the same holds for collaborations in global health. Does reliance remain a necessary and sufficient reason for collaboration in situations characterized, as are those in global health collaborations, by substantial power imbalance between partners, and what might be the role of trust in these situations?

## 5 TRUST AND RELIANCE IN ASYMMETRIC COLLABORATIONS

Collaborative research in global health has been promoted as an effective way of addressing pressing health issues that predominantly affect communities in LMIC, and also of advancing scientific research within countries where the highest burden of disease lies.^43^ As such, the implicit aim is to build capacity in LMIC to correct the 90/10 gap. Yet, scientific collaborations between HIC and LMIC have had little success, so far, in addressing the power asymmetry between HIC and LMIC.^44^

Asymmetry in HIC–LMIC partnerships is often predicated on the presumed value of what each party can contribute to the joint effort. Often, LMIC partners provide ‘raw materials’, such as knowledge and experience of the local context, relationship with relevant local communities, and samples or data. HIC partners typically process the raw materials and add value by bringing in infrastructure, technical and analytical skills, and, more often than not, the funding. By assuming one partner as a benefit‐giver and the other as a benefit‐receiver, as the capacity building aim implies, partners are positioned in asymmetric relationships. Such situations can create an asymmetry of bargaining power between the collaborators.

Consider, for example, a collaboration between a research institution from the United States, which has strong scientific and technological infrastructure and access to funding, with a research unit in Ghana, to investigate effective treatments for malaria. Malaria is prevalent in Ghana and the Ghanaian researchers contribute DNA samples and clinical data to the research project. The U.S. partner conducts the analysis of samples and data, and develops new therapeutics. The U.S. researchers hold the financial and scientific advantage and so have greater opportunities than their Ghanaian colleagues to define the rules of the collaboration. The Ghanaian researchers might feel that they have an obligation to accept the terms of the U.S. partner to get access to new and innovative malaria treatments for their population. Given the asymmetric relationship between the partners and the emerging power imbalance, both of which could undermine the possibility of a fair collaborative partnership, could reliance provide a sufficient and necessary justification for collaboration in this instance?

Relationships of reliance assume the existence of rational self‐interested agents with equal power over each other. In partnerships where symmetric power exists, distribution of risks and benefits is expected to also be symmetrical, and therefore fair. A collaboration between two researchers with the same academic standing, equal access to funding, and equal stakes in the research project can be an example of such collaboration. It is easy to imagine that these two agents would be able to negotiate the terms of the collaboration in an effective and fair way, and also stick to them, as it would be in neither's interest to do otherwise. In situations, however, where power symmetry collapses, reasons for keeping the relationship equal and fair also cease to exist. Where there is power asymmetry the stronger partner has the opportunity, and reason, to tip the balance of risk and benefits to his/her favour and at their partner's expense. Also, as it is often the case, the weak partner, in virtue of his/her position, lacks the ability to effectively punish the strong partner in case of defection, hence giving reasons to the powerful partner to drop the relationship.

Another factor that could secure a fair collaboration could be the promise of future reciprocation, i.e., a tit‐for‐tat relationship. Partners are motivated to establish a stable relationship to increase the likelihood of being granted access to the desired good/service/relationship now and in the future. Again, a partner with little to offer has less ability to motivate desire for future reciprocation. Consider the case of genomic research where the LMIC partner provides the samples and the HIC the DNA extraction, sequencing and analysis. Once the samples are handed over and genomic information extracted, these data can be used again and again for new projects without the need for the collection of new samples. There is now little dependence of the HIC partner on the LMIC partner, who would now fail to motivate the HIC partner's self‐interest to collaborate fairly. It seems, therefore, that when it comes to partnerships characterized by asymmetric power, reliance cannot reasonably justify collaboration. So, where does this leave global health research collaborations?

The preclusion of reliance as a sufficient justification for collaboration in global health research raises the question of whether trust could offer a sufficient justification. If one cannot rely on the other's self‐interest to collaborate fairly, then could trusting in one's good will offer an adequate reason for collaboration? As explained above, although trust makes the trustor vulnerable towards the trustee, as the trustee can decisively affect the outcome of the entrusted action, a reasonable belief in the trustee's good will can justify and counterbalance that risk. But trust does not, and indeed cannot, always exist. Parties in conditions of vulnerability might be forced by the circumstances to act *as if* they trust – to enter into a relationship in which the other person can decisively influence the outcome of the action – but this would be the outcome of an empty choice, rather than of a belief in the other person's good will towards them.^45^

What needs to be avoided is leaving LMIC researchers in a situation where they have no other option than to appeal to another's good will, particularly when trust has not been or cannot be established. The decision to enter an asymmetric collaborative relationship cannot in itself be seen as a proof of trust. People can behave ‘as if they trust’ in a situation where they have no other option, or where the only alternative is worse than the risk or cost associated with entering the relationship (think, for example, of a mafia boss asking you to deliver a suspicious parcel in exchange for not harming your family). One should not assume, however, that all collaborative asymmetric relationships are either irrational or coercive, nor think that trust is unimportant or irrelevant in scientific collaborations. Empirical studies have demonstrated the moderating effect of trust in asymmetric collaborations^46^ and shown that the attitude and affective disposition of a collaborator can be significant motivators in entering collaborations in global health.^47^

It is important to develop policies and create opportunities to level the playing field between HIC and LMIC, so that researchers can choose partners for collaboration based on their proven capacity and for reasons that would promote all parties’ interests. Through reliable collaborations, long‐term relationships will establish and hopefully trust will also be built between researchers and countries. However, trust relationships (whether established on real or assumed trust) should not be the only basis on which LIMC researchers could justify a collaboration. In the past few decades a number of policies and schemes have been developed by funders and other institutions to address the power imbalance problem in research, such as capacity‐building schemes built into research projects,^48^ guidelines for effective contract negotiation^49^ and funding schemes available only to researchers in developing countries.^50^ These policies aim specifically at shifting the locus of power from HIC to LMIC, and thus giving LMIC researchers the opportunity and tools necessary to enter into collaborations as equal partners. For example, the COHRED discussions of intellectual property rights and of data and sample ownership and sharing provide practical advice as to how LMIC researchers can protect themselves from becoming ‘glorified field‐workers’, demonstrate the value of their contribution, and motivate desire for future reciprocation.

I believe that the aforementioned policies and guidelines confirm my thesis, namely that reliance, rather than trust, is both necessary and sufficient for fair collaborations. LMIC researchers should have the same opportunities as their counterparts in HIC: opportunities to develop their skills, broaden their knowledge, pursue their research interests and establish themselves in the academic community. This requires not only the availability of direct funds for LMIC researchers, but also the existence of the appropriate infrastructure for the development and flourishing of research in these countries. Additionally, it requires changes at an international level to the ways in which research excellence is perceived and measured, and collaborations acknowledged in publications.^51^ Schemes have already been initiated to encourage and support LMIC–LMIC collaborations in health research.^52^ Of course, it remains to be seen whether these efforts will succeed in levelling the research arena. Empirical research can assist in this task by examining whether targets are achieved, and by revealing areas where more work is needed. However, the focus and direction towards creating the right environment for LMIC countries to pursue their research agendas and enhance their research capacity should persist.

## 6 CONCLUSION

Scientific collaborations between HIC and LMIC are an increasing trend in global health. They bring together knowledge, expertise and resources in order to find more effective ways of combating disease and illness. Collaborations between HIC and LMIC are, however, often characterized by asymmetric power distribution, making partnerships more risky and often unfair to the LMIC researchers. In this paper I have argued that in symmetric collaborations reliance, rather than trust, can provide adequate justification for entering a partnership. The term ‘collaboration’ denotes a non‐hierarchical partnership aiming at a common goal, based on reasonable expectations and proven ability. It assumes power parity between partners. In situations where power parity is not present, reliance – belief in the self‐interested motivation of the other – cannot provide adequate justification for collaboration. Trust could provide sufficient reason for collaboration in such situations, but trust relationships take time to establish, and also require one partner to become vulnerable, depending upon the other to act in a way that will not hurt or betray them. Power disparity therefore could lead to relationships where LMIC researchers are forced to act *as if they trust* HIC partners even when trust might not exist or is not reasonable.

In order to ensure that collaborations between LMIC and HIC countries are fair and equitable, the scientific playing field must be levelled. Creating the conditions in which researchers can be equal partners makes possible the formation of relationships of reliance between equal stakeholders, rather than asymmetric relationships in which the only option for the weaker partner is to trust in the other's good will.

## CONFLICT OF INTEREST

The author declares no conflict of interest.

## Footnotes

1 Parker, M., & Kingori, P. (2016). Good and bad research collaborations: Researchers’ views on science and ethics in global health research. *PLoS ONE*,* 11*, e0163579; Chu, K. M., Jayaraman, S., Kyamanywa, P., & Ntakiyiruta, G. (2014). Building research capacity in Africa: Equity and global health collaborations. *PLoS Med*,* 11*, e1001612.

2 WHO. (2017). *Global health estimates (GHE)*. Geneva, Switzerland: World Health Organization.

3 Bill and Melinda Gates Foundation. *Global Grand Challenges*. https://gcgh.grandchallenges.org/about, accessed 5 August 2017.

4 National Institute of Allergy and Infectious Diseases. (2016). *Neglected tropical diseases*. https://www.niaid.nih.gov/research/neglected-tropical-diseases, accessed 5 August 2017.

5 Research Councils UK. (2017). *Global challenges research fund*. http://www.rcuk.ac.uk/funding/gcrf/, accessed 5 August 2017.

6 Wellcome Trust. Wellcome Trust Centre for Global Health Research. https://wellcome.ac.uk/news/wellcome-trust-centres-global-health-research, accessed 5 August 2017.

7 Ad Hoc Committee on Health Research Relating to Future Intervention Options. (1996). *Summary of investing in health research and development*. Geneva, Switzerland: World Health Organization. http://apps.who.int/iris/bitstream/10665/63139/1/TDR_GEN_96.2.pdf

8 Council on Health Research for Development (COHRED). (2013). *Where there is no lawyer: Guidance for fairer contract negotiation in collaborative research partnerships*. http://www.cohred.org/wp-content/uploads/2012/04/Fair-Research-Contracting-Guidance-Booklet-e-version.pdf; Parker & Kingori, *op. cit*. note 1.

9 Boshoff, N. (2009). Neo‐colonialism and research collaboration in Central Africa. *Scientometrics*,* 81*, 413; Parker & Kingori, *op. cit*. note 1.

10 COHRED, *op. cit*. note 8.

11 Ibid: 2.

12 Ad Hoc Committee on Health Research Relating to Future Intervention Options, *op. cit*. note 7; COHRED, *op. cit*. note 8.

13 Wellcome Trust. (2015). Wellcome Trust Collaborative Awards – enabling the partnerships of the future. In *Wellcome Trust*. https://blog.wellcome.ac.uk/2015/01/20/wellcome-trust-collaborative-awards-enabling-the-partnerships-of-the-future/, accessed 5 August 2017; Medical Research Council. (2017). Global health funding partnerships. Medical Research Council. https://www.mrc.ac.uk/funding/science-areas/international-global-health-research/funding-partnerships/, accessed 5 august 2017.

14 Ibid.

15 Henneman, E. A., Lee, J. L., & Cohen, J. I. (1995). Collaboration: A concept analysis. *Journal of Advanced Nursing*,* 21*, 103–109; D'Amour, D., Ferrada‐Videla, M., Rodriguez, L. S. M., & Beaulieu, M. D. (2005). The conceptual basis for interprofessional collaboration: Core concepts and theoretical frameworks. *Journal of Interprofessional Care*,* 19*, 116–131.

16 Hord, S. M. (1986). A synthesis of research on organizational collaboration. *Educational Leadership*,* 43*, 22–26.

17 In order to demonstrate the distinction between different modes of working relationships, Mattessich, Murray‐Close and Monsey contrast collaborations with cooperation, which they define as a simple agreement between organizations which furthers their individual aims and goals, but lacks a common mission, structure or joint planning; and coordination, where two parties decide to work together in order to better achieve their independent goals. Mattessich, P. W., & Monsey, B. R. (1992). Collaboration: what makes it work. A review of research literature on factors influencing successful collaboration. Amherst H. Wilder Foundation, St Paul, Minnesota.

18 Baggs, J. G., & Schmitt, M. H. (1988). Collaboration between nurses and physicians. *Image: The Journal of Nursing Scholarship*,* 20*, 145–149; Henneman et al., *op. cit*. note 15; Hanson, C. M. (2000). Collaboration. In A. B. Manric, J. A. Spross, & C. M. Hanson (Eds.), *Advanced nursing practice. An integrative approach*. (2nd edn.) (pp. 315–347). Philadelphia, PA: W.B. Saunders.

19 Kraus, W. A. (1984). *Collaboration in organizations: Alternatives to hierarchy*. New York, NY: Human Sciences Press, p. 19; Henneman et al., *op. cit*. note 15.

20 Mattessich & Monsey, *op. cit*. note 17.

21 Kraus, *op. cit*. note 19, p. 127

22 Wu, J., & Axelrod, R. (1995). How to cope with noise in the iterated prisoner's dilemma. *Journal of Conflict Resolution*,* 39*, 183–189.

23 D'Amour et al., *op. cit*. note 15.

24 Ibid; Kraus, *op. cit*. note 19; Pike, A. W., McHugh, M., Canney, K. C., Miller, N. E., Reiley, P., & Seibert, C. P. (1993). A new architecture for quality assurance: Nurse–physician collaboration. *Journal of Nursing Care Quality*,* 7*, 1–8; Parker & Kingori, *op. cit*. note 1.

25 D'Amour et al., *op. cit*. note 15, p. 116 (emphasis added).

26 Kerasidou, A. (2016). Trust me, I'm a researcher!: The role of trust in biomedical research. *Medicine, Health Care and Philosophy*,* 20*, 43–50; Holton, R. (1994). Deciding to trust, coming to believe. *Australasian Journal of Philosophy*,* 72*, 63–76; Wright, S. (2010). Trust and trustworthiness. *Philosophia*,* 38*, 615–627.

27 Jones, K. (1996). Trust as an affective attitude. *Ethics*,* 107*, 4–25, p. 4.

28 Baier, A. (1986). Trust and antitrust. *Ethics*,* 96*, 231–260.

29 Ibid: 224.

30 Ibid.

31 Jones, K. (2012). Trustworthiness. *Ethics*,* 123*, 61–85.

32 It is important to acknowledge that trust and reliance are also relevant to institutions. A discussion of the role of trust in and reliance on institutions and the relationship between institutions in collaborative global health research would be an useful addition to the literature, but falls outside the remit of this paper.

33 Of course, this presupposes trust in the system of accountability that regulates the reliance relationship. It can be argued therefore, that reliance does exist independently, but only emerges in a system where different layers of trust relationships exist. This might be true at a systemic level; at the personal level, however, it is still valid to distinguish between relationships of reliance and relationships of trust.

34 Mouzas, S., Henneberg, S., & Naudé, P. (2007). Trust and reliance in business relationships. *European Journal of Marketing*,* 41*, 1016–1032, p. 1022.

35 Frost‐Arnold, K. (2013). Moral trust and scientific collaboration. *Studies in History and Philosophy of Science Part A*,* 44*, 301–310.

36 Adler, J. E. (1994). Testimony, trust, knowing. *The Journal of Philosophy*,* 91*, 264–275; Sztompka, P. (2007). Trust in science. *Journal of Classical Sociology*,* 7*, 211–220; Blais, M. J. (1987). Epistemic tit for tat. *The Journal of Philosophy*,* 84*, 363–375; Fricker, E. (2002). Trusting others in the sciences: A priori or empirical warrant? *Studies in History and Philosophy of Science Part A*,* 33*, 373–383.

37 Of course, there can be instances where the colleague may also be a friend whom you trust to take into account the fact that you rely on them for the completion of the project. In this case, failure to fulfil the entrusted action would justify feelings of betrayal.

38 Jones op. cit. note 31, p. 65 (emphasis added).

39 Wagner, C. S. (2005). Six case studies of international collaboration in science. *Scientometrics*,* 62*, 3–26; Parker & Kingori, *op. cit*. note 1.

40 Shrum, W., Chompalov, I., and Genuth, J. (2001). Trust, conflict and performance in scientific collaborations. *Social Studies of Science*,* 31*, 681–730.

41 Mouzas et al., *op. cit*. note 34, p. 1023.

42 Ledford, H. (2008). Collaborations: With all good intentions. *Nature*,* 452*, 682–685.

43 COHRED, op. cit. note 8.

44 Ibid.

45 It is important to distinguish the vulnerability that stems from asymmetric power from the vulnerability assumed in a trust relationship. The former is a situational property, whereas the latter is a relational one. Not all who are situationally vulnerable also become relationally vulnerable, i.e., not all people in a vulnerable situation decide to trust, and not all people who trust are also situationally vulnerable. See Kerasidou, *op. cit*. note 26.

46 Caniëls, M. C. (2009). The moderating effect of trust on the relationship between power asymmetry and organizational learning. In *OLKC, International Conference on Organizational Learning, Knowledge and Capabilities, VU University Amsterdam;* Ozlati, S. (2015). The moderating effect of trust on the relationship between autonomy and knowledge sharing: A national multi‐industry survey of knowledge workers. *Knowledge and Process Management*,* 22*, 191–205. Caniëls’ working definition of trust (‘we feel that this customer can be counted on to help us’) and Ozlati's ‘benevolence‐based trust’ are close to my definition of trust. Ozlati's competence‐based trust is closer to my definition of reliance.

47 Parker & Kingori, *op. cit*. note 1.

48 Malaria Genomics Epidemiology Network. Malaria Genomics Epidemiology Network; Bloomfield, G. S., Xavier, D., Belis, D., Alam, D., Davis, P., Dorairaj, P., … Yan, L. L. (2016). Training and capacity building in LMIC for research in heart and lung diseases: The NHLBI‐United Health Global Health Centers of Excellence program. *Global Heart*,* 11*, 17–25.

49 COHRED, *op. cit.* note 8.

50 Bill and Melinda Gates Foundation. (2017). *Grand Challenges Africa – Innovation Seed Grants*. https://gcgh.grandchallenges.org/challenge/grand-challenges-africa-innovation-seed-grants, accessed 5 August 2017; Medical Research Council. (2017). *African Research Leader scheme*. https://www.mrc.ac.uk/funding/science-areas/international-global-health-research/funding-partnerships/arl/, accessed 5 August 2017.

51 Teixeira da Silva, J. A. (2011). The ethics of collaborative authorship. *EMBO Reports*,* 12*, 889–893; Smith, E., Hunt, M., & Master, Z. (2014). Authorship ethics in global health research partnerships between researchers from low or middle income countries and high income countries. *BMC Medical Ethics*,* 15*, 42.

52 H3Africa. (2017). *Human heredity and health in Africa*. https://h3africa.org/

